# Mobility of the first metatarsal-cuneiform joint in patients with and without hallux valgus: in vivo three-dimensional analysis using computerized tomography scan

**DOI:** 10.1186/s13018-015-0289-2

**Published:** 2015-09-15

**Authors:** Xiang Geng, Chen Wang, Xin Ma, Xu Wang, Jiazhang Huang, Chao Zhang, Jian Xu, Junsheng Yang

**Affiliations:** Department of Orthopedics, Huashan Hospital, Fudan University, 12 Middle Wulumuqi Road, Shanghai, 200040 China

**Keywords:** Computed tomography, First ray, Hypermobility, Three-dimensional motion, Weight-bearing

## Abstract

**Background:**

Hallux valgus (HV) deformity is closely correlated to the hypermobility of the first metatarsal-cuneiform joint, but adequate understanding of the three-dimentional (3D) mobility of this joint in normal or HV feet is lacking. This study was conducted to investigate the mobility of the first metatarsal-cuneiform joint in multiple planes during body weight-bearing conditions for both normal and HV patients.

**Methods:**

A total of 10 female volunteers (20 feet) and 10 female HV patients (20 feet) participated in this study. Using a custom-made foot-loading device, computerized tomography (CT) scans of each pair of feet were taken under both unloaded and body weight-bearing conditions. 3D models were reconstructed for the first metatarsal and the medial cuneiform. Rotational and translational motions of the first metatarsal-cuneiform joint in multiple planes from unloaded to loaded conditions were quantitatively evaluated by reverse-engineering software.

**Results:**

During body weight-bearing conditions, the first metatarsal-cuneiform joint in HV feet dorsiflexed at an average of 2.91° (standard deviation, SD 1.71) versus 1.18° (SD 0.47) in controls (*t* = 4.158, *P* = 0.001); supinated 2.17° (SD 2.28) versus 0.98° (SD 0.81) in controls (*t* = 2.080, *P* = 0.045); and internally rotated 2.65° (SD 2.22) versus 0.96° (SD 0.57) in controls (*t* = 3.114, *P* = 0.006). Moreover, the joint in HV feet widened significantly compared with the controls (*t* = 2.256, *P* = 0.030) and tended to translate more in the dorsal-plantar direction (*t* = 1.928, *P* = 0.063); the translation in the medial-lateral direction was not significantly different between the two groups.

**Conclusions:**

During weight-loading process, the first metatarsal-cuneiform joint turns dorsiflexed, supinated, and internally rotated. For HV feet, hypermobility of the first metatarsal-cuneiform joint can be observed in multiple planes. This study promotes further understanding of the physiological and pathological mobility of the first metatarsal-cuneiform joint.

## Background

Hallux valgus (HV) deformity is closely correlated to hypermobility of the first metatarsal-cuneiform (MC) joint [1–4]. However, adequate understanding of the first MC joint mobility is limited. Several studies have been conducted to assess the mobility of this joint, but a number of difficulties were encountered, including the following. First, manual evaluation is subjective, with poor reproducibility and validity [5]. Second, device-assisted physical examination [6, 7] fails to differentiate the isolated motion of the first MC joint from the whole first ray [8]. Finally, current two-dimentional (2D) radiographic images [9–11] reflect only the motion in the sagittal plane while ignoring the potential changes on the axial or coronal plane, which may also be important in hypermobility.

With the advent of multiple-plane imaging, computed tomography (CT) can be used to reconstruct a three-dimentional (3D) model from 2D images, providing the possibility of measuring 3D kinematics between the small tarsal bones. More importantly, CT scanning under loaded conditions has recently been reported to study foot deformities [12–14], which is promising for describing the mobility of the first MC joint. Weight-bearing conditions are necessary for evaluating HV deformity [15], and body weight, compared with manually applied force, can provide the most common daily stress that induces multi-planar motion of the first MC joint.

The present study attempts to quantitatively assess the multi-axial rotation and multi-planar translation of the first MC joint during unloaded to body weight-bearing conditions and compare these motions between healthy and HV feet. This study can facilitate understanding of the physiological and pathological mobility of the first MC joint, and may inform future evaluation and treatment.

## Methods

### Subjects

This study conformed to the Declaration of Helsinki and was approved by the Ethics Committee of Huashan Hospital. The purpose, methods, and risks of the research were explained to all the potential participants. Inclusion criteria are the following: considering the overwhelming preponderance of HV in females, as well as the tendency for articular degenerative changes in the older population, subjects in this study were confined to women between 20 and 50 years old. Exclusion criteria are the following: subjects with generalized ligamentous laxity according to a 9-point scale [16] and those having feet with inflection or history of trauma or surgery were excluded.

Ten patients were randomly recruited among those who attended our department with bilateral HV deformities during January–March 2015 and consented to participate in this research. The HV deformity in this study was defined when the first metatarsal-phalangeal angle (i.e., the hallux valgus angle (HVA)) was more than 15°. Ten other patients with healthy feet were randomly enrolled among those who visited our department during the same period for upper extremity problems and consent to participate.

### Unloaded and loaded CT scan

**Fig. 1:**
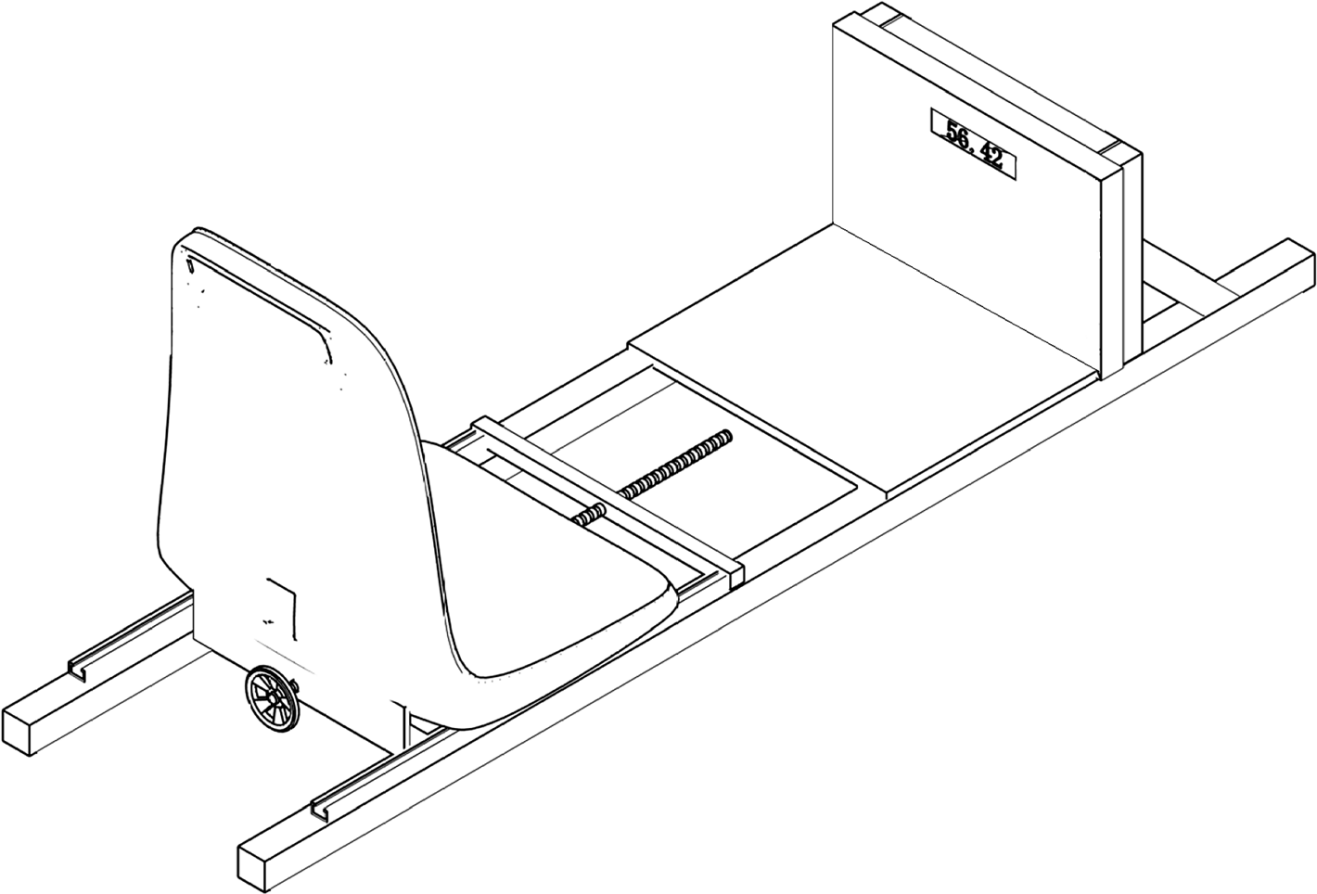
The custom-made foot-loading device. It consists of a wooden frame base, a vertical foot plate with a force-meter embedded, a seat with flanges beneath it which could slide along the slots on the frame base, and a screw-loading system

A custom-made device was used to perform CT scans for each pair of feet in both unloaded and body weight-bearing conditions. This device consisted of a frame base, a foot plate, a seat, and a screw-loading system (Fig. 1), which was similar to that reported by Ferri et al. [17]. Most of the materials were made of wood to minimize artifacts during CT scanning. The foot plate was vertical to the frame base with a height of 30 cm and could accommodate different foot sizes. A digital force meter was embedded in the plate that could withstand up to 120 kg and had good accuracy. Flanges were built beneath the seat that fit into and slide along the slots on the frame base. A screw was attached to the seat base such that the seat could be pushed forward to the foot plate by rotating the screw handle, thus exerting adjustable force through the participants’ bodies to their feet.

The participants sat upright in the seat during CT scanning. Velcro straps were used to fix their knee joints at full extension and keep their lower extremities horizontal. The ankle was positioned in neutral position, and the foot was placed with its longitudinal axis vertical to the CT bed without pronation or supination. Weight-bearing CT scan was performed by regulating the screw-loading system to make sure that the readout of the force meter is equal to the body weight of the participant, whereas unloaded CT scan was applied with the minimum possible readout.

CT images were acquired in the axial plane from the tibiotalar joint to the sole in 0.6-mm contiguous slices using a 64-slice spiral CT (100 kV × 80 mA, volume EC, 512 × 512 matrix). The images were imported into a 3D reconstruction software package (Mimics 14.1; Materialise Inc., Leuven, Belgium) in the Digital Imaging and Communication in Medicine format. The 3D models of the first metatarsal and the medial cuneiform, both in unloaded and loaded condition, were segmented and reconstructed.

### 3D mobility analysis

**Fig. 2:**
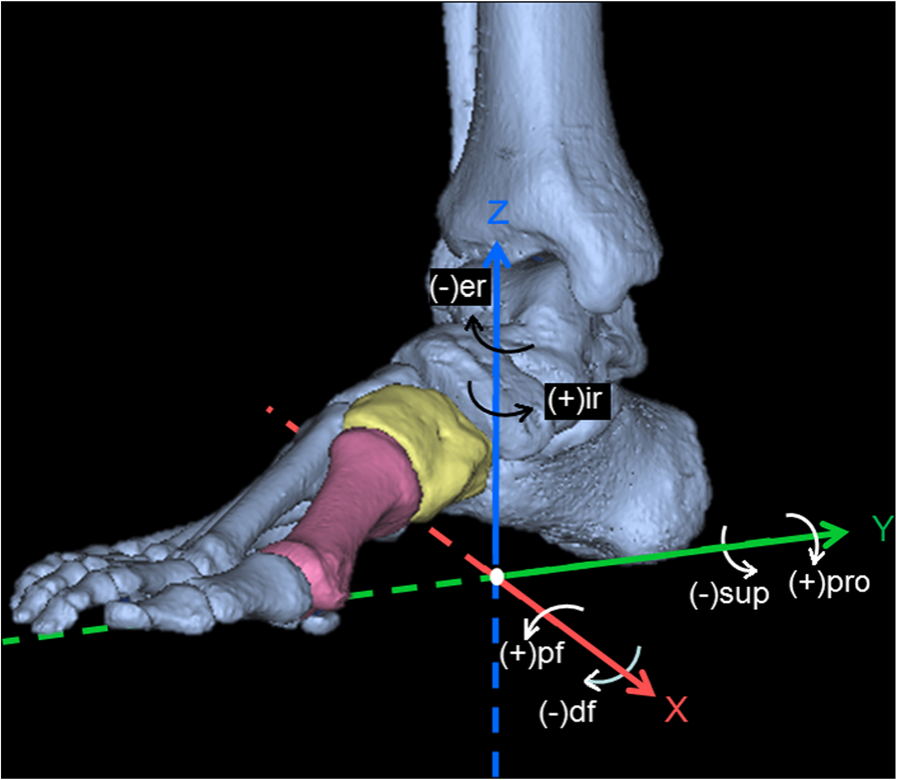
A global X–Y–Z coordinate system used to describe the motion of the first metatarsal-cuneiform joint. The Y-axis was parallel to the connecting line between the center of the heel and the second toe and pointed back; the X-axis was set perpendicular to the Y-axis and directed from the lateral aspect to the medial aspect of the foot; and the Z-axis was set perpendicular to the XY plane and pointed dorsally. According to the right-hand rule, plantar-flexion, pronation, and internal rotation were defined as positive, and dorsiflexion, supination, and external rotation were defined as negative. *pf* plantar flexion, *df* dorsiflexion, *pro* pronation, *sup* supination, *ir* internal rotation, *er* external rotation

The multi-planar motion of the first MC joint was evaluated using a reverse-engineering software package (Geomagic Studio 13.0; Geomagic Co, NC, USA). Based on the theory of rigid body mechanics, the principal axes at the centroid of the solid body were rotated with the body to determine the degree of motion, which was described using a global X–Y–Z coordinate system (Fig. 2). The Y-axis was parallel to the connecting line between the center of the heel and the second toe, pointing back; the X-axis was set perpendicular to the Y-axis and directed from the lateral aspect to the medial aspect of the foot; and the Z-axis was set perpendicular to the X–Y plane, pointing dorsally. In this study, plantar flexion, pronation, and internal rotation were defined as positive; dorsiflexion, supination, and external rotation were defined as negative.

**Fig. 3:**
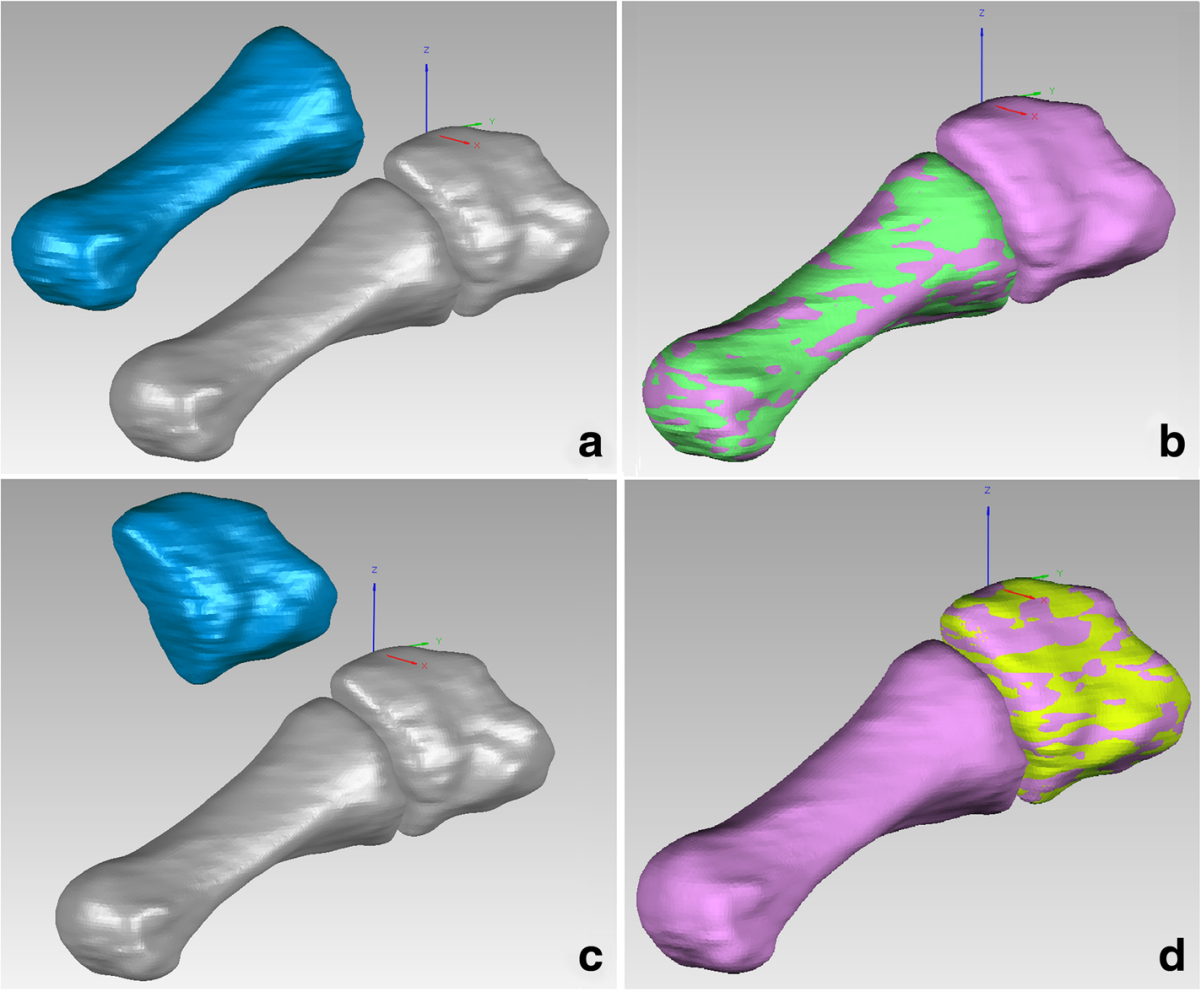
Global registration between unloaded and loaded models. **a** unloaded first metatarsal (*blue*) and loaded first metatarsal and medial cuneiform (*gray*). **b** Through adequate rotation and translation of the unloaded first metatarsal, its global registration with the loaded model was accomplished. **c** Unloaded medial cuneiform (*blue*) and loaded first metatarsal and medial cuneiform (*gray*). **d** Through adequate rotation and translation of the unloaded medial cuneiform, its global registration with the loaded model was accomplished

Both the first metatarsal and medial cuneiform in unloaded condition (i.e., the moving bone) were matched to the corresponding bones in loaded condition (i.e., the stationary target) through global registration [12, 13] (Fig. 3). During this matching process, the unloaded models of these two bones precisely moved and overlapped with their loaded targets. The software we used automatically recorded the rotational changes around different axes to demonstrate the spatial movements of the two bones from unloaded to loaded conditions. By subtracting the motion of the medial cuneiform from that of the first metatarsal, the multi-axial rotation of the first MC joint could be calculated.

Furthermore, by merging the loaded first metatarsal and cuneiform into a single model and matching its metatarsal portion with the unloaded first metatarsal, the coordinate difference between unloaded and loaded cuneiform centroid could reflect multi-planar translation of the first MC joint.

**Fig. 4:**
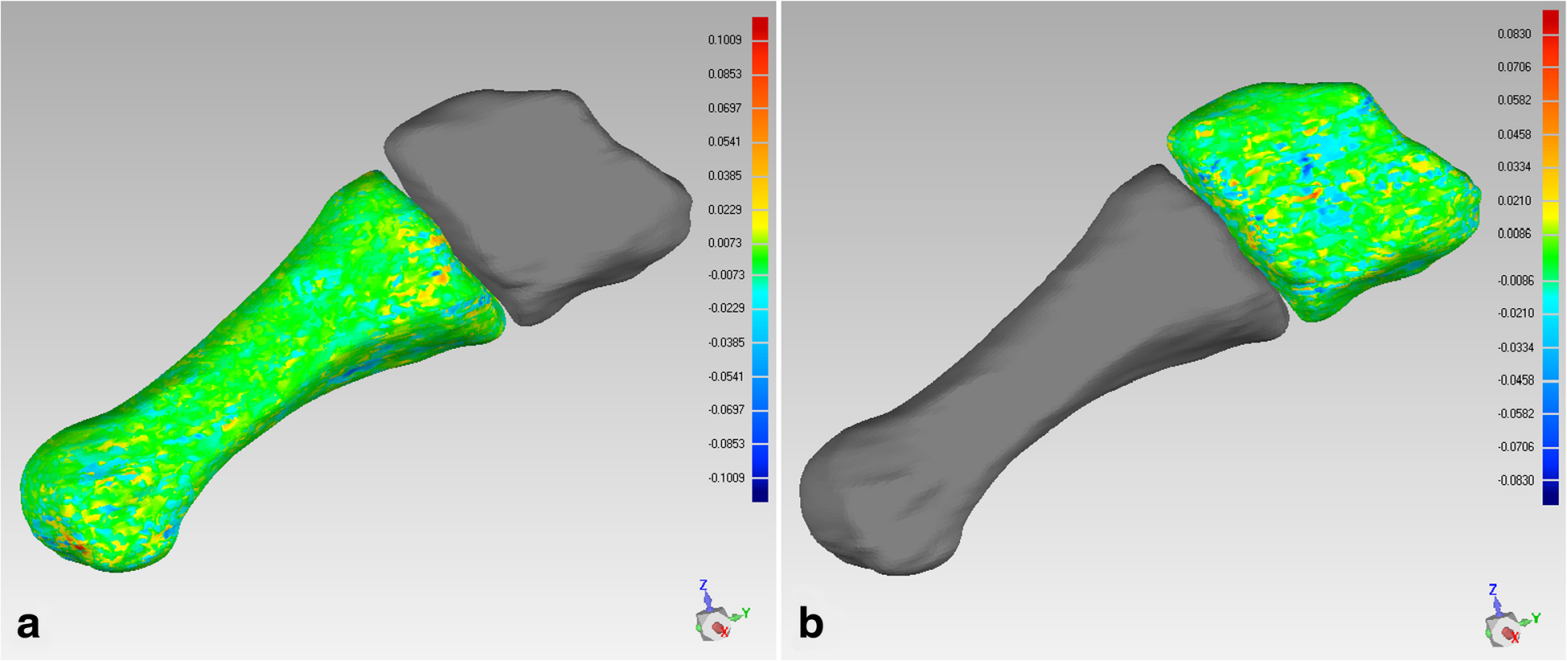
Deviation analysis to verify the accuracy of each registration. It suggested the deviation was less than 0.1 for both **a** metatarsals’ and **b** cuneiforms’ registration

The accuracy of this method was 0.1 mm in translation and 0.1° in rotation [12, 13, 18], and in this study, deviation analysis was performed to verify the accuracy of each registration (Fig. 4).

### Statistical analysis and sample size determination

The results of multi-axial rotation and multi-planar translation were presented as the mean and standard deviation (SD), and were compared between healthy and HV feet using an independent-samples *t* test. Differences with *P* values less than 0.05 were considered statistically significant. Statistical analysis was performed using SPSS 20.0 software (SPSS Inc., Chicago, USA).

Sample size was estimated for independent-samples *t* test [19]. According to previous studies [9–11] about the sagittal mobility of the first MC joint, which was also the main movement, the mean difference between healthy and HV feet was 1.8° with a SD of 1.2°. When the significance level is set at 0.05 and the statistical power is set at 0.9, a minimum of 10 cases were required for each group. Therefore, the sample size of this study, i.e., 20 healthy feet and 20 HV feet, was above the minimum requirement. The estimation procedures were performed using PASS 11 software (NCSS Inc., Kaysville, USA).

## Results

The 10 HV patients had a mean age of 38.4 ± 6.4 years old and a mean weight of 52.1 ± 5.9 kg, whereas the controls had a mean age of 35.7 ± 6.1 years old and a mean weight of 53.4 ± 6.2 kg. No significant differences in age or weight were detected between the two groups.

The mean HVA in the controls was 11.4° (SD 2.7), and the mean intermetatarsal angle (IMA) was 6.0° (SD 2.1). In the HV group, the mean HVA was 33.6° (SD 10.7), and the mean IMA was 12.5° (SD 3.2). Data on the multi-axial rotation and multi-planar translation of the first MC joint during weight loading are presented in Tables 1 and 2.

**Table 1:** Multi-axial rotation of the 1st MC joint during weight-bearing comditions

	Around X-axis (°)			Around Y-axis (°)			Around Z-axis (°)		
	pf (+)	df (−)	Mean(SD)	pro (+)	Sup (−)	Mean (SD)	Inr (+)	Exr (−)	Mean (SD)
1st metatarsal									
Healthy feet	0	20	−1.56 (0.94)*	20	0	3.03 (1.51)	20	0	1.50 (0.53)
HV feet	0	20	−3.13 (1.29)*	20	0	3.55 (1.97)	20	0	2.28 (2.06)
Medial cuneiform									
Healthy feet	4	16	−0.38 (0.69)	20	0	4.00 (1.69)*	18	2	0.54 (0.79)*
HV feet	8	12	−0.22 (1.49)	20	0	5.72 (2.04)*	8	12	−0.37 (1.01)*
1st MC joint									
Healthy feet	0	20	−1.18 (0.47)*	2	18	−0.98 (0.81)*	20	0	0.96 (0.57)*
HV feet	0	20	−2.91 (1.71)*	3	17	−2.17 (2.28)*	20	0	2.65 (2.22)*

*pf* plantar flexion, *df* dorsiflexion, *pro* pronation, *sup* supination, *inr* internal rotation, *exr* external rotation, *HV* hallux valgus, *MC* metatarsal-cuneiform

*Significant difference between two groups (*P* value <0.05)

**Table 2:** Multi-planar translation of the medial cuneiform relative to the first metatarsal during weight-bearing conditions

	Along X-axis (mm)			Along Y-axis (mm)			Along Z-axis (mm)		
	m (+)	l (−)	Mean (SD)	po (+)	an (−)	Mean (SD)	do (+)	pl (−)	Mean (SD)
Healthy feet	8	12	−0.09 (0.23)	12	8	0.17 (0.43)	0	20	−0.44 (0.35)
Hallux valgus feet	11	9	−0.01 (0.51)	17	3	0.56 (0.64)	0	20	−0.74 (0.61)
*P* value	\		0.551	\		0.030*	\		0.063

*m* medial, *l* lateral, *po* posterior, *an* anterior, *do* dorsal, *pl* plantar

*Significant difference between two groups

After weight loading, the first metatarsals of HV feet were dorsiflexed around the X-axis at a significantly larger degree than that of healthy feet. Given that the first metatarsals were more dorsiflexed than the medial cuneiforms in all feet, each of the first MC joints in both groups was in dorsiflexion, with those in HV feet presenting a significantly larger degree (Table 1).

Around the Y-axis, all the first metatarsals and medial cuneiforms pronated after weight loading. However, in most instances (18 healthy feet and 17 HV feet), the degree of pronation of the medial cuneiform was larger than that of the first metatarsal, and thus, the corresponding first MC joint was supinated. The rotational degree of the medial cuneiform and the first MC joint in the HV feet was significantly larger than that in healthy feet (Table 1).

Around the Z-axis, all the first metatarsals in both groups internally rotated after weight loading. However, external rotation of the medial cuneiform was relatively more common in the HV group. All first MC joints presented internal rotation, with the HV feet showing a significantly larger degree (Table 1).

No significant difference was observed in the translation of the first MC joint along the medial to lateral direction (the X-axis) between healthy and HV feet. However, along the Y-axis, significantly greater widening was observed in HV feet. Along the Z-axis, the joint tended to translate more with dorsal lift of the first metatarsal and plantar depression of the medial cuneiform, and the difference approached statistical significance (Table 2).

## Discussion

The axis of motion of the first MC joint is mainly in a plantar medial-to-dorsal lateral plane. Thus, most available clinical examinations, manual or special device-assisted, are performed along this axis. Klaue et al. [6] developed a handheld device to quantify first ray mobility and defined hypermobility as sagittal translation greater than 8 mm. Later, Glasoe et al. [7] designed another device that could measure first ray mobility more precisely. However, all these methods, including manual examination, could not isolate the MC joint from the first ray mobility.

Several radiological instability signs have demonstrated the increased motion of the first MC joint. King and Toolan [9] reported that the first metatarsal in HV feet lifted 2 mm on average and dorsiflexed 2° relative to the medial cuneiform based on weight-bearing lateral X-rays. Faber et al. [10] utilized the Coleman block test to magnify dorsiflexion and plantar-flexion of the MC joint and found that the mean mobility was 12.9° on lateral radiographs. Dietze et al. [20] found that the mean maximum dorsiflexion angle was 2.6° (SD 1.3) during the normal gait cycle. However, all these radiological assessments were limited to the sagittal plane.

The available studies involving multi-planar motion of the first MC joint were all performed on cadaveric specimens. Ouzounian and Shereff [21] applied reference pins and computer-assisted 3D tracking system to capture the joint motion of the normal foot and found that the mobility was 3.5° (SD 1.9) in the sagittal plane and 1.5° (SD 1.1) in the coronal plane. In another cadaveric study using 2D LED video registration [22], the mobility of the first MC joint was observed 2.4° (SD 1.6) in the sagittal plane and 2.2° (SD 0.8) in the transverse plane. However, these cadaveric studies could not reflect the real conditions in live feet.

The present study overcame these limitations and successfully evaluated the 3D motion of the first MC joint in vivo. The rotational and translational mobility in the sagittal plane are in line with previous studies mentioned earlier in this article [9, 10], but the magnitude was relatively smaller. This finding may be due to the presence of more stabilizing factors in live feet and because the motion was assessed under common body weight instead of extra forces. Furthermore, a new finding in this plane that has not been previously reported is that the first MC joint widened more in HV feet compared with healthy feet.

In the transverse plane, the rotational mobility in HV feet is in good agreement with the aforementioned study by Faber et al. [22]. Moreover, Tanaka et al. [15] reported that the IMA between the first and second metatarsals in HV group was significantly increased on weight-bearing dorsoplantar radiographs compared with non-weight-bearing view. The result of the present study further suggests that the internal rotation degree of the first MC joint in HV feet is significantly greater than that in healthy feet. However, the translation in the medial-lateral direction was not significantly different between the two groups. This observation may be because the simulated body weight could not provide sufficient force in this direction to expose the difference.

In addition to the sagittal and transverse planes, this study also quantitatively evaluated the coronal rotation, which is highly difficult to assess by routine radiographs. In previous studies, the pronation of the first metatarsal in HV feet was observed by Mortier et al. through tangential radiographs [23] and by Collan et al. through weight-bearing CT scan [24]. However, the coronal rotation of the medial cuneiform and the rotational mobility during body weight-bearing in this plane were not evaluated. In the present study, the rotational mobility in healthy feet was smaller compared with that obtained from a previous study by Ouzounian and Shereff [21]. This finding may also be related to different ways of inducing joint motion. Interestingly, most medial cuneiforms pronate more than the first metatarsal after loading both in healthy and HV feet, so that the MC joint usually turns out to be supinated, especially in HV feet. This phenomenon has never been documented in previous studies.

The highlight of our study lies in its methodology. By using a custom-made device and CT scan with thin slices, we acquired 3D bone models under non- and body weight conditions. With the aid of global registration, multi-planar motion of the joint around different axes during weight-bearing process was quantitatively assessed in vivo.

This study, however, has several limitations. First, subgroups according to different deformity magnitudes or clinical symptoms were not introduced in HV feet, and thus, the data obtained about the pathological mobility of the first MC joint are merely preliminary. Second, only the weight-bearing condition is simulated in this study, but the foot naturally changes its position throughout the day. Therefore, further analysis of the 3D mobility of the first MC joint for different kinds of HV deformity under different physiological conditions is necessary in the future.

## Conclusions

The first MC joint turns dorsiflexed, supinated, and internally rotated during unloaded to loaded conditions. These rotational motions are all significantly larger in HV feet than in healthy feet. Moreover, the joint also widens more in HV feet. Therefore, hypermobility of the first MC joint not only exists in HV deformity but also involves multiple planes. These findings promote further understanding of the physiological and pathological mobility of the first MC joint and may inform future treatment.

## Abbreviations

CT: computed tomography
HV: hallux valgus
HVA: hallux valgus angle
IMA: intermetatarsal angle
MC: metatarsal-cuneiform
2D: two-dimentional
3D: three-dimentional
